# The personal roles dimension of the theory of work and personal role reconciliation: a constructivist grounded theory study

**DOI:** 10.3389/fpubh.2025.1663762

**Published:** 2025-11-18

**Authors:** Miguel Valencia-Contrera, Lissette Avilés, Naldy Febré

**Affiliations:** 1 Faculty of Nursing, Andrés Bello University, Santiago, Chile; 2 Nursing Studies Department, University of Edinburgh, Edinburgh, Scotland, United Kingdom

**Keywords:** work-life balance, nursing, intensive care units, qualitative research, grounded theory

## Abstract

**Background:**

Although the theory of work and personal role reconciliation has been recently published, the personal roles dimension has not yet been explored in depth. This article aims to describe its theorization.

**Methods:**

Constructivist grounded theory was employed to conceptualize the phenomenon of interest from the perspectives of nurses, their families, and administrative staff. Data were derived from 202 h of non-participant observation in two high-complexity hospitals in Chile (one public and one private), 57 institutional documents, and 51 in-depth interviews. Data analysis followed the constant comparative method and multilevel coding. To ensure methodological rigor, the study adhered to the 13 criteria for constructivist grounded theory research proposed by Charmaz and Thornberg and was approved by a Scientific Ethics Committee.

**Results:**

Personal roles are defined as the set of behaviors associated with the personal context of intensive care unit nurses, which are shaped by eight sources of interaction: (1) Family dimension; (2) Extended family; (3) Community groups; (4) Recreation spaces; (5) Religious institutions; (6) Health care institutions; (7) Educational institutions; and (8) Other public services.

**Conclusion:**

The study presents the theorization process of the personal dimension involved in the reconciliation of work and personal roles among nurses in Intensive Care Units. Personal roles are essential to understanding this reconciliation process. The findings provide evidence of the existence of eight sources of interaction, which are influenced by the cultural context.

## 1 Introduction

Work–family interaction has been defined as “a process in which a worker’s functioning (behavior) in one domain (e.g., home) is influenced by (negative or positive) load reactions that have built up in the other domain (e.g., work)” (1). Formal study of this phenomenon began in the late 20th century with the development of the first theoretical models in the field (2). Since then, three main approaches have been identified (3): 1. The first views the phenomenon as a conflict, where work and family are seen as incompatible and antagonistic domains; 2. The second frames it as a matter of balance, implying the implementation of strategies or practices to harmonize or balance the demands of each domain; 3. The third approach conceives it as an interaction, where the work and family spheres influence each other bi-directionally, potentially generating both positive and negative outcomes.

The relevance of studying this phenomenon lies primarily in its numerous and varied negative associations, including impacts on child mental health (4), the quality of romantic relationships (5), marital problems (6), and parental frustration and irritability in caregiving (7). These negative associations were further exacerbated during the COVID-19 pandemic, particularly among nurses with rigid and extended work schedules, increasing stress and negatively affecting their well-being (8).

In Chile, the legal framework supports the assessment of this type of occupational risk through the Copenhagen Psychosocial Questionnaire (COPSOQ) (9, 10), with the Superintendence of Social Security (SUSESO) being the entity responsible for publishing its results. According to its latest report, the health sector workforce shows the highest percentage of suboptimal psychosocial risk levels (medium and high risk), reaching 89.7% (11).

From the state of the art, the literature has identified that among the various occupational groups in the healthcare sector, nurses are particularly vulnerable to this phenomenon (8, 12). This is due, among other factors, to the high proportion of women in the profession (13)—approximately 90%, according to WHO data (14). Within nursing practice, critical care settings have been de-scribed as environments with a heightened level of conflict between work and personal spheres when compared to general units (15–17).

Despite the importance of the phenomenon, Chauhan and Rai (18), in a recent literature review, observed that research to date has taken a rather narrow view by treating family and caregiving responsibilities as the core of the non-work domain. This aligns with Reimann et al. (19), who argue that the personal dimension of the interaction between professional and personal roles has received limited attention, despite the multiple and complex relationships within this domain. Such limitations in dimensionality may help explain inconsistent results or lack of findings, highlighting the need for a conceptual debate that transcends classical perspectives. In this regard, Mude and Wesley (20) emphasize that qualitative approaches can provide a more comprehensive understanding of the interaction between work and personal roles, which is crucial for the development of more nuanced theoretical models.

The recognition of an external source of interaction beyond the workplace—unrelated to the individual characteristics of workers—is not a recent development. Reviews dating back to 1976 already identified elements such as family problems or the death of a loved one as sources of stress that influenced employees’ work performance (21). The predominant approach in the current state of the art has focused primarily on the domains of work and family/home (18). However, literature published over two decades ago already referred to the “non-work domain” as something broader than merely home or family (22). Nevertheless, what lies outside of work continues to remain largely unexplored to this day (18).

We have recently developed the theory of work and personal role reconciliation (23), which explains the process of reconciling work and personal roles among Intensive Care Unit (ICU) nurses, based on the perspectives of the nurses themselves, their families, and nursing supervisors. Role reconciliation is described as the core category that accounts for the interaction between nurses’ work and personal roles. This process consists of three stages: resisting the war of roles, hitting rock bottom, and reconciling.

In the original publication (23), we described the central process of the theory; however, the theorization of personal roles has not yet been explored in depth. In this regard, the present study was designed to address this gap by theorizing the personal dimension involved in the work–personal role reconciliation process of intensive care unit nurses.

## 2 Materials and methods

### 2.1 Study design

A Constructivist Grounded Theory (CGT) approach was employed (24), as it is a research design that enables the uncovering of invisible social processes. This design acknowledges that data analysis is an act of interpretation, rather than an objective report or a single vision of the phenomenon under study. In this line, the researcher becomes more attuned to the relativity of the empirical world, considering multiple viewpoints and realities (25). Therefore, the priorities, values, positions, and actions of the researcher in constructivist grounded theory are not neutral, as they will shape the interpretation of the data (26).

Like the original grounded theory, CGT follows an inductive logic (27); however, it incorporates distinctive elements (24): 1. It adopts a reflexive stance toward its own background, values, actions, research contexts, relationships with participants, and representations of them; 2. It acknowledges that both researchers and participants hold multiple perspectives, roles, and realities; 3. It situates the research within its historical, social, and situational conditions of production; 4. It embraces a relativist epistemology.

Symbolic interactionism was used as the theoretical-methodological framework (28), offering a worldview and language suitable for conducting CGT studies (29). This framework made it possible to document changes within social groups and to understand the core processes associated with those changes (30).

The present study was reported following the Consolidated Criteria for Reporting Qualitative Research (COREQ) checklist (31). For further details regarding the methodological decisions of the study, readers are referred to the research protocol (32).

### 2.2 Researcher positioning

Given the constructivist stance adopted in the present study, it is essential to disclose the researcher’s positionality. The principal investigator (MVC) is a male nurse and aca-demic at a Chilean university. His clinical experience has been developed in ICU settings, including during the COVID-19 pandemic.

Regarding his academic background, his interest in occupational health and safety began during his undergraduate training and deepened during his master’s studies, with a particular focus on psychosocial risks. Among these, he developed a specific affinity for the work–family conflict, which later became the subject of his doctoral dissertation in Nursing Science. The present study is part of one of the articles derived from his doctoral thesis. Therefore, the principal investigator’s clinical experience in ICU settings, combined with his scientific training related to the phenomenon of interest, provided a unique theoretical sensitivity throughout the theorizing process.

### 2.3 Recruitment

The recruitment process was conducted in Chile, a country located on the southwest-ern edge of South America, with an approximate population of 18 million inhabitants distributed across 16 regions (33). The Chilean healthcare system has a mixed structure, meaning that both public and private institutions coexist within its healthcare network (34).

In light of the above, two institutions—one public and one private—were included in the present study and anonymized as H1 and H2. Recruitment began at H1 using purposive sampling and then continued at H2 following theoretical sampling in an iterative process.

After obtaining approval from the ethics committee, the principal investigator contacted the management of the participating institutions as well as the nursing staff coordinating the ICUs. During these interactions, the purpose and details of the study were explained. Subsequently, physical and digital copies of an informational brochure about the study were distributed through the official communication channels with the nursing staff of the respective institutions.

Those interested received a copy of the informed consent form, and their contact information (phone/email) was recorded to coordinate subsequent meetings according to their availability. In addition, upon completion of the nurses’ participation, they were asked whether they would agree to include adult members of their family nucleus in the study. If so, they were asked to contact their relatives to obtain their authorization and to share their contact information with the principal investigator, who subsequently inquired about their interest in participating in the study.

In this way, purposive sampling was carried out, which allowed for the generation of initial coding. Subsequently, theoretical sampling was employed to collect and analyze the additional data necessary until reaching saturation of the properties within each category.

As for the eligibility criteria, the study included ICU nurses, adult family members of nursing professionals, and nurse administrators. This allowed the incorporation of perspectives from the main actors involved in the process of reconciling work and personal roles among ICU nurses. Nurses who were not actively working at the time of data collection, due to medical leave or other reasons for work absence, were excluded from the study.

### 2.4 Data collection

Three data collection methods were employed: non-participant observation, in-depth interviews, and document analysis, following constructivist grounded theory strategies (35–37). Data collection began after receiving approval from the scientific ethics committees and was carried out throughout the year 2024. This process was concluded once theoretical saturation was reached, that is, when no new properties of the categories emerged from the data.

Data collection began at H1 with non-participant observations, guided by the following question: What is the process of work–family interaction among ICU nursing professionals? Field notes were taken using the format provided in (Supplementary material 1) and were later digitized for analysis. The information obtained through observations helped to identify the individual and collective actions of nurses, as well as environmental characteristics and the consequences of social behaviors (38).

In-depth interviews were informed by the observations and focused on individuals with expertise in the phenomenon of interest—namely, those who had experienced it directly or had observed others undergoing the experience (39). Accordingly, nurses who were living the interaction between work and personal spheres were selected, along with strategic actors from each domain. From the work domain, this included coworkers and nurse administrators; from the personal sphere, adult family members of the nurses were included. Interviews were conducted in locations previously arranged with participants, most of whom preferred their workplaces, where a designated room was prepared for the study. The interview guide used is available in (Supplementary material 1). Interviews lasted an average of 53 min, were audio-recorded, and transcribed for subsequent analysis. No interviews had to be repeated, and transcripts were not returned to participants, primarily because the experiences were analyzed iteratively following grounded theory strategies.

Finally, the third method of data collection consisted of document analysis. This helped to contextualize the theorization within cultural, social, economic, situational, historical, and political frameworks (37). Examples of the documents analyzed include: the Chilean Constitution (40), health laws (41, 42), labor laws (10, 43), international ILO conventions (44, 45), ministerial guidelines for the management of psychosocial risks at work (46), intersectoral guidelines from the Ministry of Health and the Ministry of Education (47), and a ministerial guide for strengthening school–family relationships (48).

### 2.5 Data analysis

Data analysis was primarily based on constant comparison and multi-level coding. The coding technique varied according to the data source: incident-by-incident coding was used for observations, line-by-line coding for in-depth interviews, and word-by-word coding for documents, following recommendations in the field (49). This process was managed and analyzed using ATLAS.ti version 25 (ATLAS.ti GmbH, Berlin, Germany).

Analysis began with the first round of data collection (50), generating an initial coding phase, followed by an iterative cycle of analysis and additional data collection. The process continued with focused coding and categorization and concluded with theoretical construction. Throughout the process, memo writing was used as a transversal strategy to record impressions, thoughts, possible directions, and emerging questions. Abductive reasoning was applied, that is, explanatory reasoning that began with concepts and concluded with the formulation of theory (51).

The category “personal roles” emerged as the core category explaining the influence of the personal sphere of workers in the process of role interaction. Repetition of patterns was used as the criterion for saturation in this study (37). Supplementary material 2 presents the coding process for the development of this category.

### 2.6 Rigor and trustworthiness

The criterion for completing the grounded theory was to ensure that the concepts developed in the theorization could be applied beyond the specific context and situation in which they were originally identified, that is, achieving “theoretical transferability” (39). This was accomplished primarily by including two institutions in participant recruitment—one public and one private—thus allowing for contextual variation. The degree of abstraction attained enabled the particular findings from H1 to be transferable to a different context (H2).

Throughout the study, CGT techniques were applied, including memo writing, constant comparison, and multi-level coding (29). Additionally, gerunds and *in vivo* codes were used to emphasize the processual nature of the phenomenon and to uncover meanings reflective of participants’ realities, with triangulation across the various data collection methods.

Finally, the study adhered to the 13 criteria of CGT rigor proposed by Charmaz & Thornberg (50). Below is a detailed account of how each criterion was met: 1. Methodological self-awareness was maintained through analytic memos, and all research decisions were explicitly justified. 2. The research project was guided by expert scholars in the topic and methodology (NF & LA), and grounded in classic literature in the field. 3. A critical literature review was conducted and used as a theoretical foundation throughout the manuscript. 4. Significant data were collected, focusing on key actors involved in the phenomenon of interest. 5. Methodological decisions were transparently documented at every stage of the study. 6. Data collection and analysis were conducted iteratively, with constant feedback between data and emerging theory. 7. Ambiguity was acknowledged and addressed through memos and reflective supervisory sessions. 8. Progressive analytical questions were formulated to support abductive reasoning (see Supplementary material 2). 9. Emerging theoretical explanations were identified and integrated into the results and discussion sections. 10. A sufficient volume and variety of data were obtained to support comparisons and sustain analytical categories. 11. Categories were continually refined, with justification of their properties and theoretical scope. 12. Codes, categories, and frameworks were treated as provisional; the theory developed is considered substantive. 13. Findings were critically discussed in relation to the current state of the art.

### 2.7 Ethical considerations

The research protocol was approved by three Scientific Ethical Committees (SECs), in accordance with the requirements of each institution where data were collected. The first approval was granted by the SEC of the Faculty of Nursing at Universidad Andrés Bello on April 3, 2024, under the code “L4/CECENF/01–2024.” Additionally, expedited reviews were conducted by the ethics committees of the participating institutions. Approval was granted on July 8, 2024, by the Scientific Ethics Committee of the Western Metropolitan Health Service under the code “10/2024” (public institution), and on August 5, 2024, by the ethics committee of the private institution under the code “162–11-24”.

Among the main ethical considerations adopted, it is worth highlighting that oral and written informed consent was obtained from all study participants. Confidentiality was ensured at all times by anonymizing participants through the assignment of a unique code.

## 3 Results and discussion

The study findings will be presented alongside the discussion, in accordance with recommendations for reporting grounded theory studies. This approach is justified as the data are analyzed in light of the state of the art and theoretical literature in the field, facilitating the articulation of the abductive reasoning process (52). The data analyzed in this study comprised 202 h of observation, 57 documents, and 51 in-depth interviews. Table 1 presents the demographic data of ICU nurses and nurse administrators, while Table 2 presents the demographic data of the nurses’ family members.

**Table 1:** Demographic data of clinical nurses (*n* = 38) and nurse administrators (*n* = 8).

Demographic data	Clinical nurses (*n* = 38)	Nurse administrators (*n* = 8)
Gender		
Male	10	0
Female	28	8
Age		
25–30	4	0
31–40	24	3
41–50	8	2
51–65	2	3
Unit*		
Intensive Care Units, adult (ICUa)	32	6
Intensive Care Units, pediatric/neonatal (ICUp)	6	2
Children/dependents under their care		
No	22	4
Yes	16	4
Work Experience (years)		
1–10	13	1
11–20	22	4
21–30	2	2
31–50	1	1
Experience in current position (years)		
1–10	31	1
11–20	6	7
21–30	1	0

*ICUa includes adult critical care units, both general and specialized (medical, surgical, burn, neurocritical, and coronary units); ICUp includes critical care units for pediatric and neonatal patients.

**Table 2:** Demographic data of nurses’ family members (*n* = 5).

Demographic data	*n*
Gender	
Male	3
Female	2
Age	
18–30	1
31–40	3
41–50	1
Occupation	
Worker	4
Student	1

**Figure 1:**
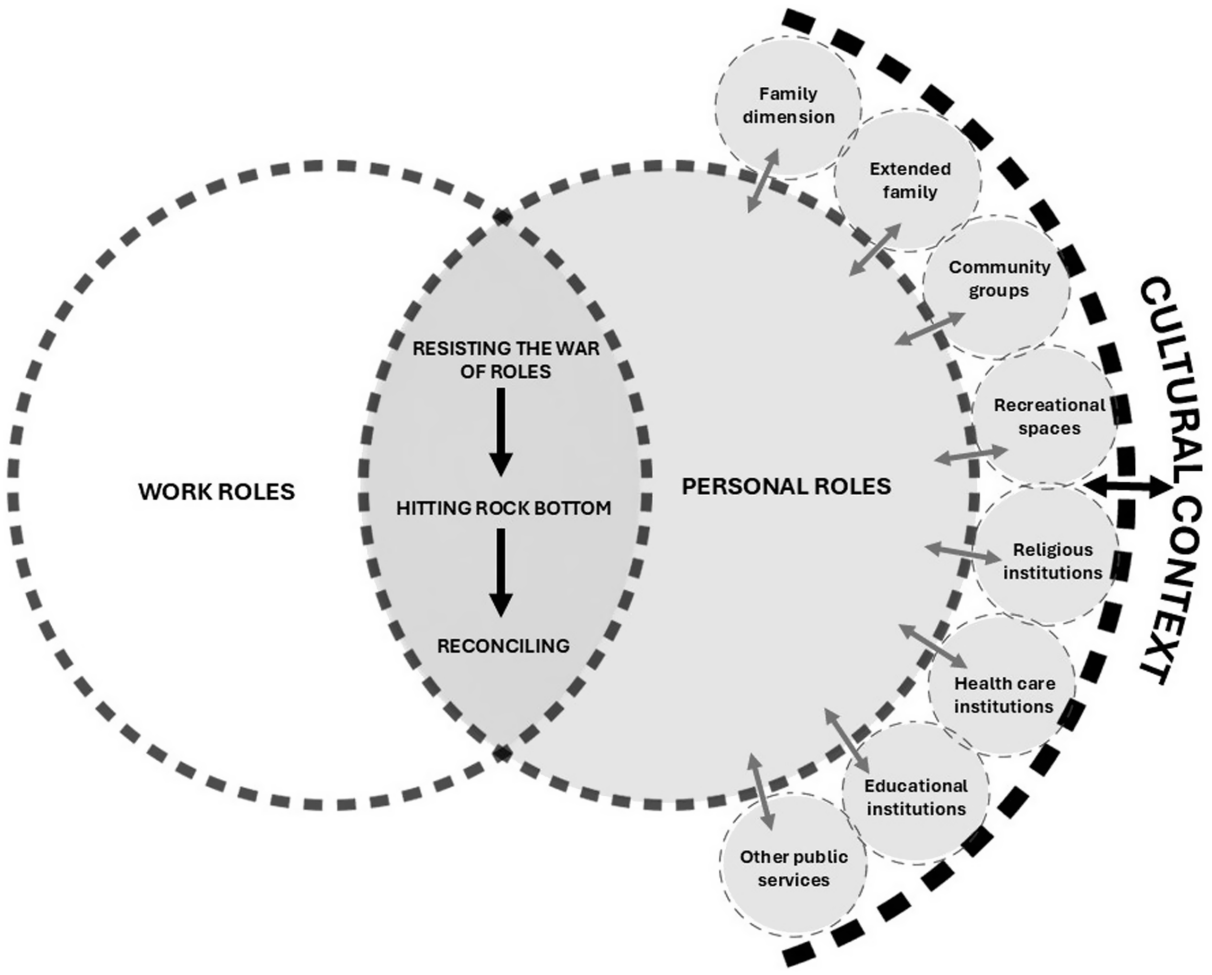
Personal roles dimension of the theory of work and personal role reconciliation.

Work and personal role reconciliation is understood as a process composed of three stages: “resisting the war of roles,” “hitting rock bottom,” and “reconciling.” These stages explain the transition experienced by a worker until achieving the reconciliation of their roles. Nevertheless, this process involves sources of interaction associated with each role; that is, there are sources of interaction linked to workers’ work roles and sources of interaction linked to their personal roles. In our first study, we published the basic social process (23); in our second study, we explained the sources of interaction of work roles (53); and finally, in the present article, we explain the personal roles (see Figure 1).

Personal roles are defined as the set of behaviors associated with the personal context of ICU nurses—an adaptation of Biddle’s definition of roles (54). The data from this study revealed that nurses’ behaviors within their personal sphere were shaped by eight sources of interaction (see Figure 1): 1. Family dimension; 2. Extended family; 3. Community groups; 4. Recreational spaces; 5. Religious institutions; 6. Health care institutions; 7. Educational institutions; and 8. Other public services.

These sources are, in turn, permeated by the cultural context (55). Thus, the family should be understood as one among several social subsystems. To fully comprehend the phenomenon, an analysis of its relationship with the other social subunits is essential; without this, the phenomenon would be understood only partially.

### 3.1 Family dimension

Based on Quintero Velásquez’s definition, the family is the matrix of identity and the psychosocial development of its members (56). In this sense, it must adapt to society and ensure the continuity of its corresponding culture. There are various definitions of family, which may be grounded in biological, structural, functional, or subjective criteria (57). In this study, following a subjective criterion, participants described a wide range of perceived definitions of family. One of the most frequently mentioned definitions referred to people who live in the same household, as illustrated by the following participant:

“*Unfortunately, one can be a bit selfish, wanting to spend time only with their partner, and unfortunately, you have to include the family—all the people, everyone who lives under your roof*”. (Interview, Female nurse 1, ICUa, H1)

In other cases, family was not associated with cohabiting members, but rather with conjugal relationships, as noted by Nurse 7:

“*I don’t have a family of my own yet; I live with my parents… but I’m already thinking about moving in with my partner*”. (Interview, Female nurse 7, ICUa, H1)

However, the concept of family was not limited to people—it also extended to animals and plants:

“*…my dogs are my children, for me, they are my children. They give me something that humans don’t, and for them, I give everything, everything*”. (Interview, Female nurse 19, ICUa, H2)

“*We also have plants at home, and when I’m not there—just like with my dogs—my father-in-law takes care of them, because sometimes I work 24-hour shifts...*” (Interview, Male nurse 15, ICUa, H1)

In yet other cases, the workplace itself was considered family, as expressed by Nurse 22:

*Interviewer MVC [author]: Who do you consider part of your family? Nurse 22: “For me, my family right now is my job, my place, the circle of friends I have, and the environment I live in...*” (Interview, Male nurse 22, ICUa, H2)

These various conceptions of family form the basis for understanding the “family dimension” as a source of interaction within the theorization of work–personal role reconciliation. This is primarily due to the different roles a person may assume within this dimension, as responsibilities, concerns, and support vary according to its members—many of whom require care. This phenomenon is referred to as double-duty caregiving, that is, healthcare workers who also serve as unpaid caregivers in their homes (58). The theory of negotiating professional–familial care boundaries by Ward-Griffin et al. (59) describes how nurses negotiate the boundaries between professional and familial caregiving in their lives. This process is characterized by constant change and shaped by the interaction of expectations, resources, and negotiation strategies. These findings align with the present study, where the family unit acted either as a facilitator or a barrier in the process of reconciling work and personal roles (see Figure 1).

As a facilitating source, the family dimension provided organized support for managing responsibilities, emotional assistance during work-related stress, and instrumental help in daily functional and logistical aspects:

“*My family is full support... they message me during shifts when I’ve had bad days. I’ve been through really dark times during my shifts, but it’s all about my mood—I think no one really notices, but it’s like carrying around a little gray cloud, and everything becomes very difficult. Then, if you’re in a good mood, you have the exact same shift, and it feels so much easier. So, during those depressive periods—I’ve had two—there’s my family. They message me, I feel their support, the bed is made when I get home, they send me food...*” (Interview, Female nurse 7, ICUa, H1)

“*…he’s my support [referring to her partner], he’s asleep, but if something happens at 2 a.m., I message him, I send a voice note. I know he’ll listen when he wakes up and reply, but at least I’ve vented. Instead of creating a problem, I just tell him what happened—and that’s how I feel I’m letting it out… on weekdays he listens right away*”. (Interview, Female nurse 33, ICUp, H1)

As a hindering source, the family dimension imposed a reproductive workload managed from the workplace, health-related issues among family members, and dysfunctional relationships:

“*It’s like they [the nurses] don’t disconnect from home—they’re still worrying, ‘Did you take their temperature? Has the diarrhea returned?’—like their mind is still at home, and yes, I’ve noticed that quite a bit*”. (Interview, Male nurse 20, ICUa, H2)

“*I’ve known many people who, for instance, have had issues with their partners and had to leave work because they weren’t emotionally well. If you think about it, that’s the foundation for being able to care for others—your family needs to be okay...*” (Interview, Female nurse 9, ICUa, H2)

These findings are consistent with the current state of the art. For example, Reimann’s review highlights that studies on work–family conflict report associations with marital and family satisfaction, family functioning, parenting behaviors, and child well-being (19). These findings emphasize not only the role of the worker but also that of family members and the family as a unit. However, family members have been identified not only as barriers to reconciliation but also as facilitators. For example, in the review by Steiner & Krings (60), the authors support the idea that positive experiences at work can transfer to one’s partner, thereby enhancing their well-being and family functioning.

### 3.2 Extended family

The extended family refers to individuals with whom the worker is related but who are not part of the nuclear family; they are people the worker does not live with on a daily basis but perceives as part of a support network. The data revealed a wide variety of individuals included in this source of interaction, such as parents, siblings, or in-laws. Like the nuclear family, the extended family functioned either as a facilitator or an obstacle in the process of reconciling work and personal roles.

As a facilitating source, the extended family provided emotional and practical support in daily life, as illustrated by Nurse 10:

“*When I go to my parents’ house, I stay for a couple of days, then return to work and feel like I’ve rested. I come back happy, cheerful, with a different kind of energy*”. (Interview, Female nurse 10, ICUa, H2)

In this sense, contact with extended family members may generate positive emotions that contribute to personal well-being and are therefore perceived as a facilitating factor in the process of reconciling work and personal roles. Additionally, the active involvement of extended family members represents an instrumental support in situations that might otherwise generate conflict, enabling nurses to better manage the demands of their multiple roles, as noted by Nurse 21:

“*One time I got a call from the kindergarten saying they had to close because the water was cut off. Thankfully, my in-laws were able to pick up my son, so I could stay on shift*”. (Interview, Female nurse 21, ICUa, H2)

However, participation from this source of interaction is conditioned by several factors such as geographic distance, the age or health status of grandparents, or whether siblings themselves are parents—situations in which reciprocal support is more common (61).

On the other hand, the extended family can also act as an obstacle, particularly when they generate demands that interfere with work responsibilities. This occurs, for example, during crises or family emergencies in which a relative requires immediate and prioritized attention, leading to interruptions in the workday and forcing the individual to prioritize personal roles, as described by Nurse Administrator 4:

“*My sister’s dog had been hospitalized after a stroke. I was on shift, and my sister called me crying—he had gone into cardiac arrest and died. He was like her baby. So I tried to calm her down and told my supervisor, ‘This is happening—I need to leave’. And she said, ‘No problem’. So I left*” (Interview, Nurse Administrator 4, ICUa, H2)

Bronfenbrenner’s bioecological perspective on human development has been described as one of the most influential frameworks for understanding how individuals relate to dynamic environments. Literature reviews have demonstrated its utility in analyzing the complex interactions between individuals and their contexts across various cultural settings (62). Initially, Bronfenbrenner developed a framework focused on ecological systems, which was later refined into the Process–Person–Context–Time (PPCT) model (63, 64).

According to this theoretical proposal, individuals are influenced by microsystems, mesosystems, exosystems, and macrosystems. The microsystem includes close environments such as family, school, peer groups, and work, while the mesosystem encompasses the interrelations among these microsystems (62). In this sense, the data from the present study align with Bronfenbrenner’s theory; however, this research adds empirical insight into how microsystems relate to one another in the context of work–personal role reconciliation among nurses.

### 3.3 Community groups

Community groups refer to collective organizations that share a symbolic or geographical space and common interests, such as parent associations at schools, neighborhood councils, and similar structures. This source of interaction was perceived as a facilitator, similar to the extended family, functioning as a form of instrumental support.

“*Fortunately, we managed to create a WhatsApp group of parents from the school. So, when I’ve been called by the school and the person who was supposed to pick up my daughter couldn’t make it, I’ve had to ask a parent I know is still at the school—and thankfully, they’ve always helped. But yes, it’s happened to me, and it definitely affects me a lot here [at work]*”. (Interview, Female nurse 23, ICUa, H2)

These findings are consistent with the consensus of the Working Time Society (65), which recommends considering neighborhood and community resources, as they can benefit child development and reduce stress for parents working shifts. These resources function as support networks by assisting with childcare during working hours and providing before- and after-school services. Without access to such community-based sources of interaction, workers often resort to less reliable childcare options or may be unable to carry out their work responsibilities. According to the literature, family is typically the primary resource when available. However, in cases where family cannot be relied upon, non-family adults, particularly close friends and acquaintances made through school environments, frequently provide support (61).

On the other hand, work roles can negatively affect this source of interaction. For example, the inherently social nature of nursing work may influence whether individuals choose to engage with members of their community groups:

“*At least in my case, being in contact with so many people—after a while, it gets tiring. Sometimes, I’m interacting with so many people at the hospital, talking to so many, having to coordinate with so many, that afterward I just don’t want to see anyone. So sometimes, for example, I don’t want to meet up with friends because I’ve already had so much social interaction here that my social battery is drained...*” (Interview, Female nurse 12, ICUa, H2)

This influence is supported by prior studies. For instance, Allan et al. (66) found that workers who are less satisfied with their work–life balance often feel too tired to enjoy their time outside of work, which in turn interferes with their social lives.

### 3.4 Recreational spaces

Recreational areas represent a source of interaction perceived as a form of distraction or leisure. The data revealed that this source was regarded as a facilitator, and participants described a wide range of recreational spaces such as gyms, restaurants, cinemas, and more.

“*On the other hand, I’m studying—I'm pursuing a second degree in psychology. So, my time is very limited. But I always try to make room for both family time and personal time. It requires a massive amount of organization, but it can be done. Sometimes it means going out to eat, having a drink, going to the movies. I’m always trying to create those moments, because if you don’t make time for recreation, you end up completely burned out*” (Interview, Female nurse 9, ICUa, H2)

“*When I work night shifts, I have time during the day. I wake up, follow my routine, go to the gym to do whatever—there’s no specific routine. I go swimming, I might exercise, but it’s mostly for my mind—I do it for my mental health*” (Interview, Female nurse 33, ICUp, H1)

These findings are supported by the current literature. For example, in the study by Chang & Bae (67), it was demonstrated that exposing working mothers to leisure activities in natural settings—distinct from their home or work environments—effectively mitigates the negative effects of work–life conflict and enhances positive emotions. Similarly, the use of strength-based therapeutic recreation has been shown to reduce conflicts between the work and personal domains of employees (68).

This is further explained by the socioecological framework linking access to green spaces with health outcomes (69), which describes how exposure to green environments generates benefits for both physical and mental health. However, these benefits are influenced by various factors, including demographics (such as gender, ethnicity, and socioeconomic status), living context, type of green space, and climate.

### 3.5 Religious institutions

Religious entities constitute a source of interaction composed of organizations associated with religious belief systems. The data from this study indicated that this source functioned as a means for developing socioemotional skills that were highly useful in professional performance.

“*In my youth, I was always involved in Christian education, in church, in social outreach activities. So for me, talking to a patient or attending to a family member is not difficult at all. Even dealing with complaints is not complicated for me, because I can connect empathetically with people—it’s very easy for me to care for someone… in this position, that helps a lot. I don’t get upset easily; it has to be something really offensive for me to get angry or scold someone. Otherwise, I’m always approachable: ‘Hey, why did you do this? What happened to you?’ Or if it’s a frustrated family member, 'Tell me, how can I help you?’ So somehow I turn the situation around and solve it, and I think that comes from this social environment I told you about, where I was raised as a young person*” (Interview, Nurse Administrator 6, ICUa, H2)

The behavior observed in this study aligns with the findings of Henderson (70), whose research concludes that religious attendance and religious support can have significant implications for well-being. Religious congregations offer a fertile ground for the development of social support, providing love, encouragement, hope, tools, and resources to cope with and make sense of problems. Moreover, studies that examine cultural differences in happiness or satisfaction among people who care for others in suffering have emphasized the role of religion. In some societies, religion enables individuals to be naturally compassionate, generous, and kind-hearted (71).

### 3.6 Health care institutions

Health care institutions represent a source of social interaction that includes professionals who provide health services. In the personal sphere, that is, outside the work setting, this source of interaction was perceived by workers as a valuable resource, offering tools to cope with the challenges of reconciling work and personal roles—such as psychotherapy:

“*I think that in the times we’re living in, with everything we see on a daily basis, psychotherapy is one of the guides we should follow—without question. Especially those of us who are more involved with patients… we should always have support*” (Interview, Female nurse 38, ICUp, H2)

“*There’s always going to be something—some case or situation that leaves a mark. In fact, during the last or second-to-last session with my psychologist, we talked about a death I experienced many years ago. I always remember it because it was a really bad shift—three patients died that night...*” (Interview, Male nurse 32, ICUa, H1)

These findings are consistent with the current state of the art. Mindfulness-based interventions have been shown to be effective in significantly reducing work–life conflict among nurses (72). Likewise, in other occupational groups, such as primary school teachers, psychotherapeutic approaches like Rational Emotive Behavior Therapy have demonstrated significant effects on well-being scores among workers experiencing work–family conflict (73).

### 3.7 Educational institutions

Educational institutions constitute a source of social interaction involving professionals who provide educational services. This source was associated with parental responsibilities and demands, such as attending parent–teacher meetings, school events, or ceremonies, which often conflicted with nurses’ work schedules due to their rotating shifts.

“*At school, when my daughter was younger, the teachers knew I couldn’t attend the class meetings. I used to tell them, ‘You always schedule the meetings when I’m on shift’… and what’s sad is that you miss activities, graduations, and so on*”. (Interview, Female nurse 36, ICUp, H1)

“*…some of my colleagues arrive a bit late because they have to drop off their kids at school*”. (Interview, Male nurse 15, ICUa, H1)

These findings are consistent with analyses in the field of family–school relationships, which describe that participation in parent–teacher interviews is not always feasible—often due to the influence of work responsibilities among a large proportion of parents (74). The influence of this source of interaction—educational institutions—has also been emphasized in the literature. For instance, the study by McCredie et al. (75) concluded that parents’ work increases parental irritability, which in turn is negatively associated with adolescents’ academic outcomes—especially among low-income mothers.

### 3.8 Other public services

Other public services represent a source of social interaction involving institutions that provide essential services to workers, such as those related to commerce, financial services, public safety, transportation, and social development. According to the data, this source of interaction was perceived as the least relevant by participants due to its occasional influence. Nonetheless, it does affect time management and, consequently, the reconciliation of roles. For example, Nurse Administrator 1 stated:

“*…I have to get home and be a mother, a daughter, a partner, myself—plus do the laundry, go grocery shopping, and get the car’s technical inspection done*” (Interview, Nurse Administrator 1, ICUp, H1)

Similarly, Nurse 2 identified other responsibilities associated with this source of interaction:

“*I arrive at work very early, I have breakfast, do the grocery shopping on my phone and have it delivered to my house, and I also pay the bills—so I can stay organized and reduce my stress*”. (Interview, Female nurse 2, ICUa, H1)

This source of interaction is acknowledged by Chilean labor law, which grants six protected days per year for public employees to be absent from work for personal matters—known as *administrative leave* (76). However, this benefit does not apply to all workers, but only to those employed in the public sector. For the rest of the workforce, employers are not legally obligated to grant such leave. In this regard, recognizing the importance of having protected time off for activities such as renewing identification documents, obtaining a passport or driver’s license, or handling banking and financial matters, a legislative proposal is currently under discussion in Chile to amend the existing regulatory framework and extend this benefit to the entire working population (77).

### 3.9 Cultural context

The cultural context refers to the learned, shared, and transmitted knowledge about the values, beliefs, norms, and ways of life of a particular group. This knowledge is typically passed down from generation to generation and shapes thinking, decision-making, and actions in a structured way (78). The data from this study reflected participants’ perceptions of the influence of Chilean culture on the behavior of the phenomenon. Participants indicated that caregiving responsibilities were distributed according to gendered patterns deeply embedded in the local context.

“*In general, in Chilean culture, it’s the mother who is always responsible for solving things. For instance, here in the unit, we have men who have children, but they never miss work when their children are sick, because it’s the mother who stays home from work to care for them*”. (Interview, Nurse Administrator 6, ICUp, H1)

*Interviewer MVC [author]: What are your thoughts on the relationship between work and family life? What’s the first thing that comes to mind? Nurse 13: “I think as a relationship per se, it’s hard to define because it depends on many contexts—it depends on your family context, on your sociocultural context, on the reality in which you live...*” (Interview, Male nurse 13, ICUa, H2)

The findings are consistent with Connell’s relational theory of gender (79), one of the most influential frameworks in the field (80, 81), which posits that gender is not fixed prior to social interaction but is instead constructed through it (82). Connell describes the set of gendered arrangements at the institutional level as the *gender regime*, and when these arrangements are part of broader patterns, they form the *gender order* of a society. These gendered arrangements comprise a network of relationships—that is, the ways in which individuals, groups, and organizations are connected and divided (83).

Gender relations are constantly being created and re-created in everyday life; however, individuals are not free to construct gender arbitrarily, as gendered practices are shaped by the gender order in which people live (83). Thus, gender is not merely an individual attribute but a social structure closely tied to culture (84). In the current grounded theory, this powerful relationship between cultural context and the phenomenon is represented through dashed lines, explicitly illustrating its cross-cutting influence on the process (see Figure 1).

Moreover, literature reviews in the field have noted that studies incorporating a gender perspective suggest that men and women experience role conflict differently, presumably due to differences in their work and family commitments or in their perceptions of work–family conflict (19). Additionally, the relationship between culture and the phenomenon has been described as significant, given that living in different cultural environments implies different approaches to resolving the issue of work–family conflict (6).

### 3.10 Strengths and limitations

Regarding the limitations of the study, these are mainly associated with the sociocultural context under investigation. Therefore, the results may be primarily applicable to contexts with similar characteristics. These limitations are consistent with what has been reported by the scientific community in studies analyzing productivity patterns related to the phenomenon between 1988 and 2021, which highlight the influence of shifting political, structural, and cultural contexts at the international level on the behavior of the phenomenon (19). In this regard, it is recommended that future research explore the applicability and theoretical scope of the findings in diverse contexts.

Finally, concerning the strengths, this article explored the personal dimension of the process of reconciling personal and work roles, addressing a knowledge gap previously noted by the scientific community, as supported by recent reviews in the field (18, 19). While the emerging theory represents only an initial proposal, it constitutes a step forward in understanding the phenomenon and provides a foundation for future research in the area.

The findings of our study provide valuable information for recognizing the sources of personal interaction involved in the phenomenon, enabling decision-makers to identify the intervening elements for subsequent management. In this way, the theory of work and personal role reconciliation represents an advancement in the study of the “work–family” conflict, offering insights into the procedural behavior of the phenomenon and the importance of considering the various work and personal sources involved in its interaction. Without such consideration, the phenomenon may be only partially addressed, which may help explain inconsistencies or unexpected results reported by the scientific community.

## 4 Conclusion

The article fulfilled its stated objective by theorizing the personal dimension involved in the process of work–personal role reconciliation among ICU nurses. The personal dimension is defined as the set of behaviors associated with the personal context of ICU nurses, shaped by eight sources of interaction: 1. Family dimension; 2. Extended family; 3. Community groups; 4. Recreational spaces; 5. Religious institutions; 6. Health care institutions; 7. Educational institutions; and 8. Other public services.

These sources are, in turn, influenced by the cultural context. The theorization of personal roles is essential for understanding the process of reconciling work and personal roles among ICU nurses.

## Data Availability

The raw data supporting the conclusions of this article will be made available by the authors, without undue reservation.
