# Supplementary material for: The personal roles dimension of the theory of work and personal role reconciliation: a constructivist grounded theory study
Source: Front Public Health. 2025 Nov 18;13:1663762. doi: 10.3389/fpubh.2025.1663762 (PMC12671553; doi:10.3389/fpubh.2025.1663762)
Supplement: Supplementary file 1 [file Supplementary_file_1.docx]

Supplementary Material 1

***DATA COLLECTION FORM: NON-PARTICIPANT OBSERVATION***

Non-Participant Observation

Date: ____ / ____ /202__ Time: _________

Location: _________________________________________________________________________

| **Observation Specification** | **Description** |
| --- | --- |
| Individual and collective actions. |  |
| Detailed notes, including anecdotes and observations. |  |
| Emphasis on significant processes occurring in the environment. |  |
| Consideration of what participants define as interesting and/or problematic. |  |
| Attention to participants' language use. |  |
| Contextualization of actors and actions within scenes and settings. |  |
| **Work charts: schemes, diagrams, and maps** | |
|  | |

***DATA COLLECTION FORM: IN-DEPTH INTERVIEWS***

**Date: ____ / ____ /202__ Participation Code: ______________________**

| **Sociodemographic Aspects** | |
| --- | --- |
| Would you like to be referred to by a pseudonym? |  |
| What gender do you identify with? |  |
| What is your date of birth? |  |
| Do you have children or family members under your care? |  |
| Considering all the jobs you have had throughout your life; how long have you been working in total? |  |
| How long have you been working in your current unit (job)? |  |

| **GUIDING QUESTIONS** | | |
| --- | --- | --- |
| **Nurses** | **Family Members of Nurses** | **Nurse Administrators** |
| -How is your routine on a day when you have to go to work?  -What are your thoughts on the relationship between work and family life?  -Tell me about a situation where you believe your work has influenced your family life.  -Tell me about a situation where you believe your family life has influenced your work. | -How is the routine on a day when your relative has to go to work?  -What are your thoughts on the relationship between work and family life?  -Tell me about a situation where you believe your relative's work has influenced family life.  -Tell me about a situation where you believe family life has influenced your relative's work. | -What are your thoughts on the relationship between work and family life of nursing staff?  -Tell me about a situation where you believe the work of nurses has influenced their family life.  -Tell me about a situation where you believe the family life of nurses has influenced their work. |
| *Notes*  ______________________________________________________________________________________________________________________________________________________________________________________________________________________________________________________________________________________________________________________________________________________________________________________________________________________________________________________________________________________________________________________________________________________________________________________________________________________________________________________________________________________________________________________________________________________________________________________________________________________ | | |
