# Supplementary material for: The personal roles dimension of the theory of work and personal role reconciliation: a constructivist grounded theory study
Source: Front Public Health. 2025 Nov 18;13:1663762. doi: 10.3389/fpubh.2025.1663762 (PMC12671553; doi:10.3389/fpubh.2025.1663762)
Supplement: Supplementary file 2 [file Supplementary_file_2.docx]

Supplementary Material 2: coding process for the development of “personal roles”

| **Data** | **Initial Codes (n=112)** | **Theoretical Links** | **Category and Focused Codes (n=22)** |
| --- | --- | --- | --- |
| *"Article 1: All persons are born free and equal in dignity and rights. The family is the fundamental unit of society".* (Document, Political Constitution of the Republic of Chile) | Personal Roles | What elements of the worker's personal dimension are involved in the interaction? What are their characteristics?  Does the theory of negotiating professional and familial care boundaries explain the participants’ experiences?  Does the relational theory of gender explain the behaviors associated with the phenomenon of interest?  What role does culture play? | -Cultural Context  -Sources of Interaction for Personal Roles: Family dimension; extended family; community groups; recreational spaces; religious institutions; health care institutions; educational institutions; other public services |
| *"My family is totally supportive... they text me during my shift when I’m going through rough times. I’ve had some very dark periods during shifts, but everything depends on my mood. I think no one notices, but for me it’s like carrying a little gray cloud—it makes everything really hard. And then, when you’re in a good mood and you have the exact same shift, it feels easy. So during the periods of depression I’ve experienced—there have been two—my family is there. They text me, I feel their support, I get home and the bed is made, they send me food..."*. (Interview, Nurse 7, UPCa, H1) |  |  |  |
| *"When I go to my parents’ house, I stay for a couple of days, and then I return to work feeling like I actually rested—I come back happy, cheerful, with a different kind of energy too"*. (Interview, Nurse 10, UPCa, H2) |  |  |  |
| *"Fortunately, a WhatsApp group of school parents was created. So, the times I’ve been called from the school and the person who was supposed to pick up my daughter didn’t show up, I’ve had to ask another parent—someone I know is still at school—and they’ve always responded, thankfully. But yes, it has happened, and that really affects me a lot here [at work]"*. (Interview, Nurse 23, UPCa, H2) |  |  |  |
